# Galectin-9 as an indicator of functional limitations and radiographic joint damage in patients with rheumatoid arthritis

**DOI:** 10.3389/fimmu.2024.1419676

**Published:** 2024-06-18

**Authors:** Jiewen Guo, Xiaoyuan Ai, Baixue Jia, Xiaoling Zhong, Lixiong Liu, Qiu Hu, Jingyi Xie, Xiaoping Hong, Yulan Chen, Dongzhou Liu

**Affiliations:** ^1^ The Second Clinical Medical College, Jinan University, Shenzhen, Guangdong, China; ^2^ Department of Rheumatology and Immunology, Shenzhen People’s Hospital, The Second Clinical Medical College, Jinan University, Shenzhen, Guangdong, China; ^3^ Department of Rheumatology and Immunology, Shenzhen People’s Hospital, The First Affiliated Hospital, Southern University of Science and Technology, Shenzhen, Guangdong, China; ^4^ Department of Radiology, Shenzhen People’s Hospital, The Second Clinical Medical College, Jinan University, Shenzhen, Guangdong, China; ^5^ Department of Radiology, Shenzhen People’s Hospital, The First Affiliated Hospital, Southern University of Science and Technology, Shenzhen, Guangdong, China

**Keywords:** galectin-9, rheumatoid arthritis, disease activity, functional limitation, radiographic joint damage

## Abstract

**Background:**

Previous studies have revealed that Galectin-9 (Gal-9) acts as an apoptosis modulator in autoimmunity and rheumatic inflammation. In the present study, we investigated the potential role of Gal-9 as a biomarker in patients with rheumatoid arthritis (RA), especially as an indicator of functional limitations and radiographic joint damage.

**Methods:**

A total of 146 patients with RA and 52 age- and sex-matched healthy controls were included in this study. Clinical data including disease activity, physical function, and radiographic joint damage were assessed. Functional limitation was defined as the Stanford Health Assessment Questionnaire (HAQ) disability index >1. Subjects with joint erosion >0 or joint space narrowing >0 were considered to have radiographic joint damage. Serum Gal-9 levels were detected by an enzyme-linked immunosorbent assay. Univariate and multivariate logistic regression analysis were used to evaluate the association between Gal-9 and high disease activity and functional limitations, and a prediction model was established to construct predictive nomograms.

**Results:**

Serum levels of Gal-9 were significantly increased in patients with RA compared to those in healthy controls (median 13.1 ng/mL vs. 7.6 ng/mL). Patients with RA who were older (>65 years), had a longer disease duration (>5 years), longer morning stiffness (>60mins), elevated serum erythrocyte sedimentation rate and C-reactive protein, and difficult-to-treat RA had significantly higher Gal-9 levels than those in the corresponding control subgroups (all p <0.05). Patients with RA were divided into two subgroups according to the cut-off value of Gal-9 of 11.6 ng/mL. Patients with RA with Gal-9 >11.6 ng/mL had a significantly higher core clinical disease activity index, HAQ scores, Sharp/van der Heijde modified Sharp scores, as well as a higher percentage of advanced joint damage (all p<0.05) than patients with Gal-9 ≤11.6 ng/mL. Accordingly, patients with RA presenting either functional limitations or radiographic joint damage had significantly higher serum Gal-9 levels than those without (both p <0.05). Furthermore, multivariate logistic regression analysis showed that a serum level of Gal-9 >11.6 ng/mL was an independent risk factor for high disease activity (OR=3.138, 95% CI 1.150–8.567, p=0.026) and presence of functional limitations (OR=2.455, 95% CI 1.017–5.926, p=0.046), respectively.

**Conclusion:**

Gal-9 could be considered as a potential indicator in patients with RA, especially with respect to functional limitations and joint damage.

## Introduction

Rheumatoid arthritis (RA) is a chronic systemic autoimmune disease that can cause impaired joint function and a high risk of disability (1). RA has an incidence of 0.5%–1.0%, with 50% of patients facing permanent disability interfering with return to work between 2 and 3 years after diagnosis (1, 2). Effective monitoring is critical for controlling joint damage and improving quality of life in patients with RA.

Galectin-9 (Gal-9), a carbohydrate-binding protein that belongs to the galectin family, is one of the ligands of T cell immunoglobulin and mucin domain 3 (Tim-3) that acts as a critical immune checkpoint (3). Gal-9 is an apoptosis-inducing factor in malignancies, autoimmune diseases, and inflammatory diseases (3–5). Gal-9 negatively regulates T cell immunity by inducing apoptosis of T helper 1 (Th1) and T helper 17 (Th17) cells (6, 7). Down-regulation of the Gal-9/Tim-3 pathway can interfere with the Th1 response and blunt apoptosis of CD4^+^ T cells in patients with RA (8). In contrast, Gal-9/Tim-3 interaction is reported to upregulate the cytotoxic activity of CD8^+^ T cells by Tim-3-expressing dendritic cells (DCs) (9) and induce maturation of DCs (10). Gal-9 also mediates neutrophil capture and adhesion in a CD44- and β2 integrin-dependent manner in the inflamed vasculature of patients with RA (11). These findings suggest that Gal-9 plays a key and complicated role in the pathogenesis of RA through different receptors.

Previous clinical studies have revealed a significant association between Gal-9 and disease activity in patients with RA. Wiersma et al. have reported that serum Gal-9 levels were elevated and were positively correlated with disease activity in patients with RA (12). Fujita et al. reported that serum Gal-9 levels increased in patients with RA and were positively associated with RA disease activity in patients with low titers of anti-cyclic citrullinated peptide antibody (ACPA; <200 U/mL) (13). Nielsen et al. also found elevated levels of Gal-9 in plasma and synovial fluids of patients with RA. However, Gal-9 levels were not correlated with long-term disease activity or radiographic progression (14). Furthermore, patients with low disease activity had significantly higher levels of Gal-9 mRNA than patients with moderate to high disease activity (15). Therefore, the role of Gal-9 in the disease activity of RA is complex and controversial, and further studies are needed to clarify the implications of Gal-9 in patients with RA.

Previous clinical studies have mainly focused on investigating disease activity rather than physical activity or radiographic joint damage in patients with RA. Therefore, in the present study, in addition to disease activity, our objective is to explore the potential role of Gal-9 as an indicator of functional limitations and radiographic joint damage in patients with RA.

## Methods

### Study patients and controls

A total of 146 patients with RA were included in the study between April 2019 and July 2021 from those attending the Department of Rheumatology and Immunology of the Shenzhen People’s Hospital, China. Patients with RA aged over 18 years who fulfilled the revised 1987 ACR criteria (16) or the criteria of 2010 ACR/European League Against Rheumatism (EULAR) (17), and completed clinical evaluations were eligible for this study. The exclusion criteria were as follows: comorbidities with other connective tissue diseases, vital organ failure, serious infections, active tuberculosis, syphilis, acquired immune deficiency syndrome, malignancy, pregnancy, and lactation. Fifty-two sex- and age-matched healthy controls (HCs) were recruited during the same period. Ethical approval for this study was provided by the Ethics Committee of the Shenzhen People’s Hospital, China (NO.: LL-KY 2022158-01). All subjects provided their informed written consent for the collection and processing of blood samples for clinical research.

### Clinical data collection

The following demographic and clinical data were collected at baseline (18): age, sex, disease duration, duration of morning stiffness, medication history within recent six months, 28-joint tender joint count (28TJC) and 28-joint swollen joint count (28SJC), patient and provider global assessment of disease activity (PtGA and PrGA, range 0–10), pain visual analogue scale (Pain VAS, range 0–10), erythrocyte sedimentation rate (ESR, 0–20 mm/h), C-reactive protein (CRP, 0–5 mg/L), rheumatoid factor (RF, 0–20 IU/mL), and ACPA (0–5 IU/mL). Disease activity was evaluated with a simplified disease activity index (SDAI), clinical disease activity index (CDAI), disease activity score in 28 joints with four variables including CRP (DAS28-CRP), and the disease activity score in 28 joints with four variables including ESR (DAS28-ESR) (19). All these indicators of disease activity were divided into four categories according to the criteria of the ACR (20): DAS28-ESR/CRP, remission with a score of <2.6; low disease activity with a score of ≥2.6 to <3.2; moderate disease activity with a score of ≥3.2 to <5.1; and high disease activity with a score of ≥5.1. In this study, difficult-to-treat RA (D2T RA) was also evaluated according to the previously reported definition (21): DAS28-ESR > 3.2 after receiving treatment with three or more disease-modifying anti-rheumatic drugs (DMARDs), one of which was biological DMARDs (bDMARDs) for a duration exceeding 18 months.

### Functional status assessment

The Stanford Health Assessment Questionnaire (HAQ) disability index, which consists of 20 items covering eight categories (dressing, rising, eating, walking, hygiene, reaching, gripping, and activities), was used to assess patient physical activity function (22). Functional limitation was defined as HAQ >1 (23). The severity of physical activity function in patients with RA was identified according to the Steinbrocker functional classification, which contains four grades based on the severity of self-reported functional impairment (24).

### Radiographic assessments

The Sharp/van der Heijde modified Sharp score (mTSS) (25) and the Steinbrocker radiographic stage (24) were used to assess structural damage in the hands and wrists by two experienced observers who were blinded to clinical data. Subjects with joint erosion (JE) >0 or joint space narrowing (JSN) >0 were considered radiographic joint damage. Advanced joint damage was defined as stage II–IV (13).

### Detection of Gal-9 in serum

Serum samples from patients with RA and from HCs were obtained at the beginning of the study and stored at -80°C until analysis. Serum Gal-9 concentrations were measured using an enzyme-linked immunosorbent assay kit (ELISA, R&D Systems, Minneapolis, USA) according to the manufacturer’s instructions.

### Statistical analysis

Statistical analyses were performed using SPSS v.25.0 software, R version 4.2.3, and GraphPad Prism software v.9.0. Continuous variables are presented as medians with their interquartile range, while categorical variables are presented as numbers and proportions (%). The Student’s t-test or the Mann–Whitney U test was used to compare continuous variables between groups. The chi-square test or Fisher’s exact test was used to compare categorical variables between groups. Spearman’s correlation analysis was performed to investigate the correlation between serum Gal-9 levels and clinical indexes. The diagnostic performance of serum Gal-9 in RA was evaluated using receiver operating characteristic (ROC) curves, and the area under the curve (AUC) was calculated using SPSS. Univariate and multivariate logistic regression analyses were performed, and the prediction model was established to draw the nomogram. A two-tailed p <0.05 was considered statistically significant.

## Results

### Baseline characteristics

The demographics and clinical characteristics of patients with RA and of the 52 sex- and age-matched HCs are shown in **Table 1**. A total of 86.3% (126/146) of patients with RA were women, with a median age of 53 years and a disease duration of 6.3 years.

**Table 1:** Baseline characteristics of patients with RA and healthy controls.

Characteristics	RA(n=146)	HC(n=52)	P value
Demographics			
Age, years, median (IQR)	53.0 (43.0-65.3)	51.0(43.3-57.5)	0.157
Gender, female, n (%)	126 (86.3)	45 (86.5)	0.966
Duration, years, median (IQR)	6.3 (2.0-15.0)		
TMS, minutes, median (IQR)	3.0 (0-30.0)		
Smoking, n (%)	12 (8.2)		
Clinical disease activity indicators, median (IQR)			
TJC28	7.0 (3.0-13.3)		
SJC28	3.0 (1.0-6.0)		
PtGA	4.5 (3.0-7.0)		
PrGA	4.5 (3.0-6.0)		
Pain VAS	4.0 (3.0-6.0)		
HAQ	0.6 (0.3-1.4)		
DAS28-ESR	5.0 (4.0-6.3)		
DAS28-CRP	4.6 (3.6-5.5)		
SDAI	22.4 (14.8-32.8)		
CDAI	19.0 (13.8-31.0)		
Laboratory data			
ESR (mm/h), median (IQR)	37.5 (23.8-68.3)		
CRP (mg/L), median (IQR)	13.5 (3.8-32.0)		
RF (IU/ml), median (IQR)	86.8 (29.8-218.9)		
RF positive, n (%)	117 (80.1)		
ACPA (U/ml), median (IQR)	102.5 (2.9-200.0)		
ACPA positive, n (%)	108 (74.0)		
X-ray of hands joints, median (IQR)			
JE subscore	25.5 (5.0-69.3)		
JSN subscore	16.0 (7.0-44.0)		
mTSS	42.0 (13.8-111.3)		
Treatment, n (%)			
Glucocorticosteroids	55 (37.7)		
csDMARDs	85 (58.2)		
Methotrexate	62 (42.5)		
Leflunomide	30 (20.5)		
Hydroxychloroquine	21 (14.4)		
Iguratimod	23 (15.8)		
bDMARDs	15 (10.3)		

28TJC, 28-joint tender joint count; 28SJC, 28-joint swollen joint count; PtGA, patient global assessment of disease activity; PrGA, provider global assessment of disease activity; Pain VAS, pain visual analogue scale; HAQ, Stanford health assessment questionnaire disability index; DAS28-CRP, disease activity score in 28 joints with four variables including CRP; DAS28-ESR, disease activity score in 28 joints with four variables including ESR; CDAI, clinical disease activity index; SDAI, disease activity was assessed with simplified disease activity index. ESR, erythrocyte sedimentation rate; CRP, C reactive protein; RF, rheumatoid factor; ACPA, Anti-citrullinated protein antibody; JE, joint erosion; JSN, joint space narrowing; mTSS score, Sharp/van der Heijde score; csDMARDs, conventional synthetic disease-modifying anti-rheumatic drugs; bDMARDs, biological disease-modifying anti-rheumatic drugs.

The proportion of patients positive for RF and ACPA was 80.1% and 74%, respectively. Elevated levels of ESR (median 37.5 mm/h, IQR [23.8–68.3]) and CRP (median 13.5 mg/dL, IQR [3.8–32.0]) were observed in patients with RA, with a median DAS28-CRP of 4.6 and a median HAQ disability index of 0.6. Furthermore, the radiographical evaluation index, including the JE subscore, the JSN subscore, and the mTSS, was also evaluated in patients with RA, with a median mTSS of 42. There were 58.2% and 10.3% of patients with RA using conventional synthetic (cs) and biological (b) DMARDs, respectively. In particular, D2T RA patients accounted for 15.1% of the included patients with RA.

### Comparison of serum Gal-9 levels in patients with RA

**Figure 1:**
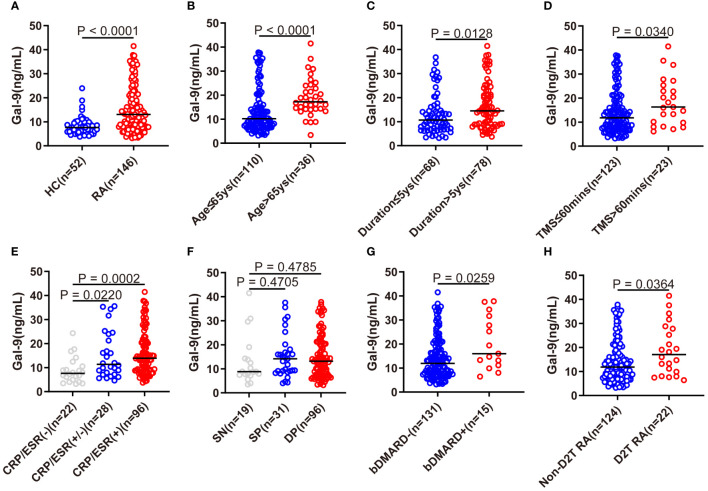
Comparison of serum Gal-9 levels in patients with RA with different clinical characteristics. **(A)** Serum Gal-9 levels in patients with RA and healthy controls (HCs). **(B–D)** Serum Gal-9 levels in patients with RA stratified by older age (age > 65 years), longer disease duration (duration >5 years), longer morning stiffness (TMS >60mins). Serum Gal-9 levels in patients with RA according to **(E)** elevated serum ESR and/or CRP, **(F)** seropositive ACPA and/or RF, **(G)** bDMARDs treatment, and **(H)** D2T RA. Gal-9, galectin-9; TMS, time of morning stiffness; ESR, erythrocyte sedimentation rate; CRP, C reactive protein; SN, seronegative; SP, semi-seropositive; DP, double-seropositive; bDMARDs, biological disease-modifying anti-rheumatic drugs; D2T RA, difficult to treat rheumatoid arthritis.

As shown in **Figure 1A**, the serum Gal-9 levels of patients with RA were significantly higher than those of HCs (median 13.1 ng/mL vs. 7.6 ng/mL, p <0.0001). We also compared the serum levels of Gal-9 in the subgroups of patients with RA with different clinical and demographic features. Patients with RA who were older (>65 years), had a longer disease duration (>5 years) and a longer morning stiffness (>60mins) had significantly higher Gal-9 levels than the corresponding control subgroups (all p <0.05) (**Figures 1B–D**).

A significantly lower serum Gal-9 level was observed in patients with RA with normal levels of ESR and CRP than in patients with elevated levels of ESR and CRP (p=0.0002), and in patients with RA with elevated levels of ESR or CRP (p=0.022, **Figure 1E**). However, there were no significant differences between patients with SN (seronegative RF and ACPA), SP (semi-seropositive RF or ACPA), and DP (double-seropositive RF and ACPA) (**Figure 1F**). In particular, compared with patients not receiving bDMARDs therapy, patients with RA on bDMARDs therapy exhibited elevated serum Gal-9 levels (p=0.0259, **Figure 1G**). Furthermore, a significantly elevated Gal-9 level was also found in D2T RA patients than in those without D2T (non-D2T) RA (P=0.0364, **Figure 1H**).

### Association of serum Gal-9 level with RA disease activity

**Figure 2:**
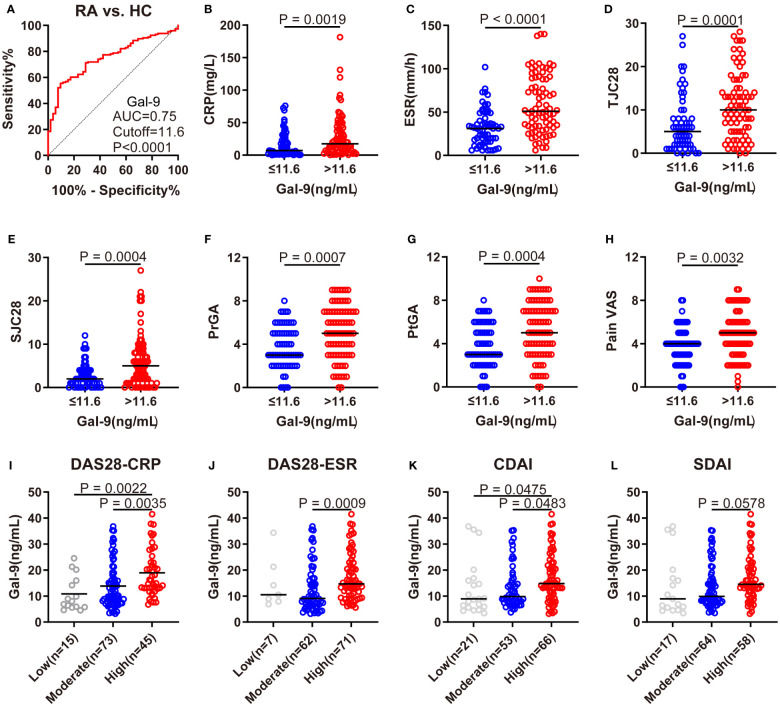
Association of serum Gal-9 levels with RA disease activity. **(A)** ROC analysis showing the performance of Gal-9 in the diagnosis of RA (cutoff value: 11.6 ng/mL). **(B–H)** Comparisons of CRP, ESR, 28TJC, 28SJC, PtGA, PrGA, and Pain VAS between subgroups. **(I–L)** Comparison of serum Gal-9 levels in patients with RA with different levels of disease activity according to DAS28-CRP, DAS28-ESR, CDAI and SDAI scores. Gal-9, galectin-9; CRP, C reactive protein; ESR, erythrocyte sedimentation rate; 28TJC, 28-joint tender joint count; 28SJC, 28-joint swollen joint count; PtGA, patient global assessment of disease activity; PrGA, provider global assessment of disease activity; Pain VAS, pain visual analogue scale; DAS28-CRP, disease activity score in 28 joints with four variables including CRP; DAS28-ESR, disease activity score in 28 joints with four variables including ESR; CDAI, clinical disease activity index; SDAI, disease activity was assessed with simplified disease activity index.

Using ROC analysis, at a cut-off level of >11.6 ng/mL, serum Gal-9 levels showed the best sensitivity (56.1%), specificity (90.4%), and an AUC of 0.75 to discriminate RA from HCs (**Figure 2A**). According to the cut-off value, patients with RA were divided into two subgroups: individuals with Gal-9 ≤11.6 ng/mL and those with Gal-9 >11.6 ng/mL. Comparisons of RA disease activity indicators between subgroups are illustrated in **Figures 2B–H**. Compared with patients with RA with Gal-9 ≤11.6 ng/mL, patients with RA with Gal-9 >11.6 ng/mL had statistically higher RA disease activity indicators, including CRP, ESR, 28TJC, 28SJC, PtGA, PrGA, and Pain VAS. Notably, a similar trend was observed when the cut-off value of 10.7 ng/mL for serum Gal-9 levels (AUC: 0.71) was adopted for identifying RA patients with DAS28-CRP ≤5.1 from those with DAS28-CRP>5.1 (**Supplementary Figure S1A**). Compared with patients with RA with Gal-9 ≤10.7 ng/mL, patients with Gal-9 >10.7 ng/mL also had significantly higher RA disease activity indicators (**Supplementary Figures S1B–H**). Furthermore, patients with RA with high disease activity had significantly higher serum Gal-9 levels than those with low or moderate disease activity in DAS28-CRP and CDAI (**Figures 2I–L**).

**Figure 3:**
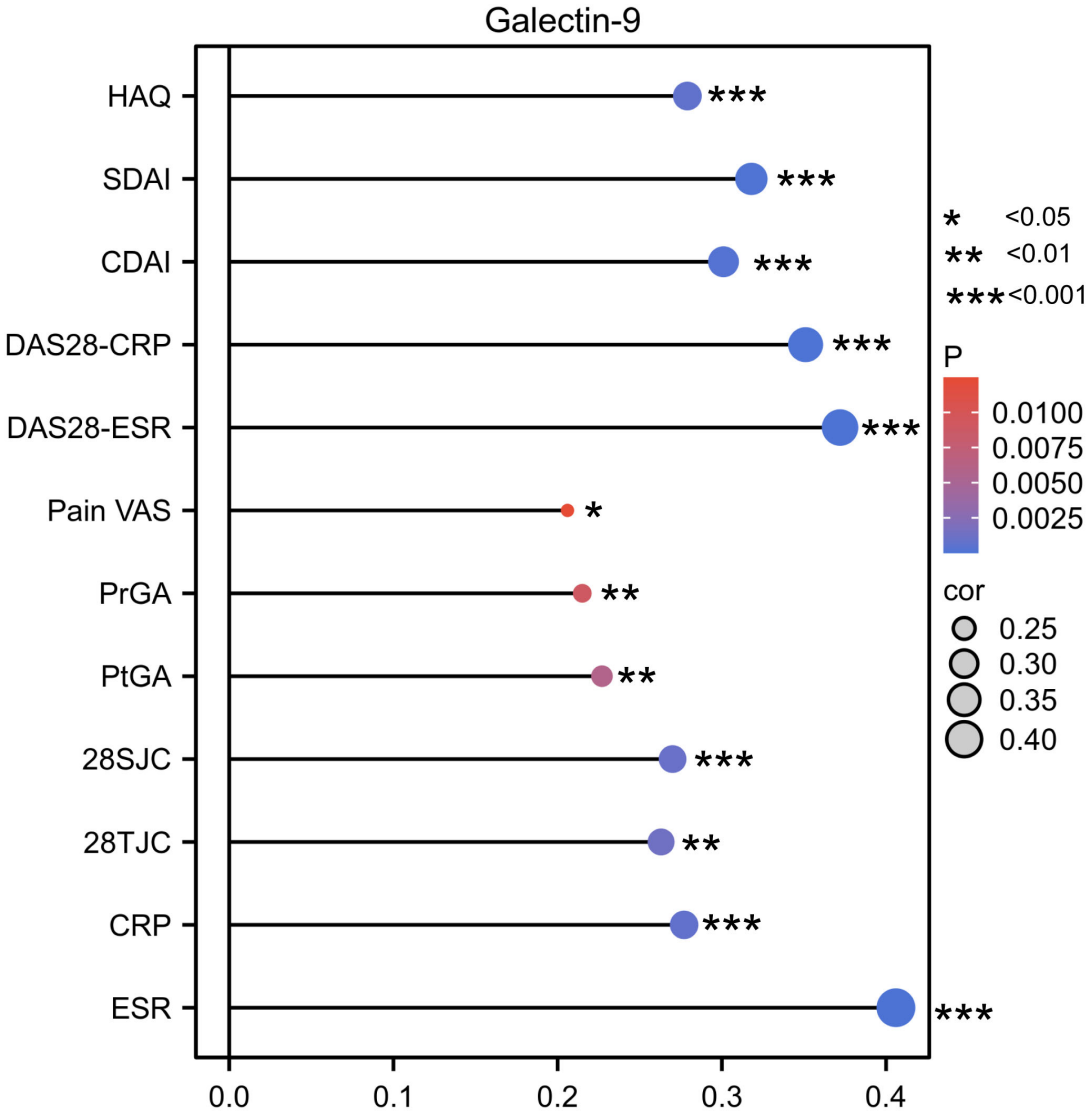
Lollipop chart showing the correlation between serum Gal-9 levels and disease activity indicators. *P < 0.05, **P < 0.01, ***P < 0.001. Gal-9, galectin-9; CRP, C reactive protein; ESR, erythrocyte sedimentation rate; 28TJC, 28-joint tender joint count; 28SJC, 28-joint swollen joint count; PtGA, patient global assessment of disease activity; PrGA, provider global assessment of disease activity; Pain VAS, pain visual analogue scale; DAS28-CRP, disease activity score in 28 joints with four variables including CRP; DAS28-ESR, disease activity score in 28 joints with four variables including ESR; CDAI, clinical disease activity index; SDAI, disease activity was assessed with simplified disease activity index.

Accordingly, as shown in **Figure 3**, serum Gal-9 levels were positively correlated with ESR (rs=0.41, p <0.0001), CRP (rs=0.28, p= 0.0007), 28SJC (rs=0.27, p=0.001), 28TJC (rs=0.26, p=0.0013), PtGA (rs=0.23, p=0.0058), PrGA (rs=0.22, p=0.009), Pain VAS (rs=0.21, p=0.0125), and HAQ (rs=0.29, p=0.0004) scores. Furthermore, serum Gal-9 was also significantly correlated with DAS28-CRP (rs=0.35, p <0.0001), DAS28-ESR (rs=0.37, p <0.0001), CDAI (rs=0.30, p=0.0002) and SDAI (rs=0.32, p <0.0001). These results suggest that serum Gal-9 was a potential marker of disease activity in patients with RA.

### Association of serum Gal-9 level with functional limitations

**Figure 4:**
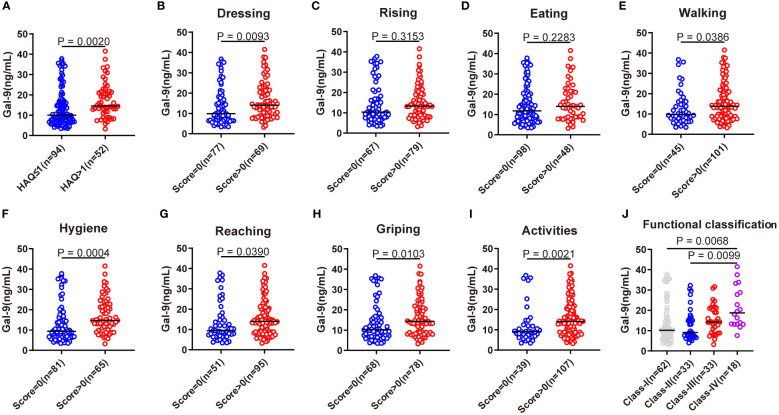
Association of serum Gal-9 levels with functional limitations. **(A)** Comparison of serum Gal-9 levels in patients with RA with HAQ ≤1 and HAQ >1. Comparison of serum Gal-9 levels in patients with RA **(B–I)** stratified by eight categories of HAQ and **(J)** according to the Steinbrocker functional classification. Gal-9, galectin-9; HAQ, Stanford health assessment questionnaire disability index.

To further evaluate the relationship between serum Gal-9 and functional limitations in patients with RA, we compared serum Gal-9 levels in different HAQ disability index subgroups and its eight physical activity function categories (dressing, rising, eating, walking, hygiene, reaching, griping, and activities). The results revealed serum levels of Gal-9 were significantly higher in patients with RA with HAQ disability index >1 than those with HAQ ≤1 (**Figure 4A**). Consequently, eight physical activity functions were divided into two groups based on whether there was a functional limitation in patients with RA. Compared with patients without functional limitations, patients with functional limitations showed significantly higher levels of Gal-9 at most physical activity function categories (all p <0.05) except for the categories of rising and eating (**Figures 4B–I**). Further analyses showed that patients with RA with Steinbrocker functional classification Class-IV had a higher level of serum Gal-9 than those with Class-I and Class-II (**Figure 4J**). These results suggest that Gal-9 was a functional limitation marker in patients with RA.

### Association of serum Gal-9 level with radiographic joint damage

**Figure 5:**
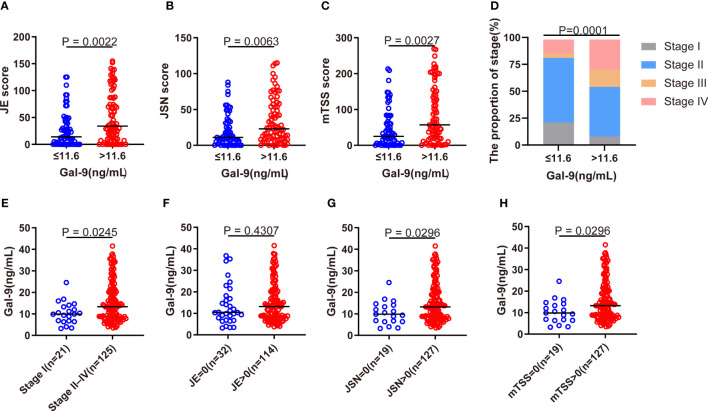
Association of serum Gal-9 levels with radiographical joint damage. **(A–C)** Comparison of JE and JSN subscores, and of mTSS scores between Gal-9 ≤11.6 ng/mL and Gal-9 >11.6 ng/mL groups. **(D)** Comparison of the Steinbrocker radiographic stage stratified by Gal-9 ≤ 11.6 ng/mL and Gal-9 >11.6 ng/mL groups. Comparison of serum Gal-9 levels in RA according to the presence of **(E)** advanced joint damage (stage II–IV) and **(F–H)** elevated JE, JSN, and mTSS. Gal-9, galectin-9; JE, joint erosion; JSN, joint space narrowing; mTSS score, Sharp/van der Heijde score.

The modified Sharp score and Steinbrocker stage were used to further evaluate radiographic joint damage in patients with RA. Compared with patients with Gal-9 ≤11.6 ng/mL, significantly higher levels of JE subscore, JSN subscore, and mTSS were observed in patients with Gal-9 >11.6 ng/mL (all p <0.05; **Figures 5A–C**). A significantly higher proportion of patients with Gal-9 >11.6 ng/mL had advanced Steinbrocker radiographical stage (II–IV) (p=0.0001; **Figure 5D**). Accordingly, a significantly elevated level of serum Gal-9 was found in patients with advanced joint damage (stage II–IV) than those without (stage I) (**Figure 5E**). Furthermore, compared with patients with RA with elevated JSN subscore and mTSS, serum Gal-9 levels were significantly lower in patients with normal JSN subscore and mTSS, respectively (**Figures 5F–H**). These results suggest that Gal-9 was a radiographical joint damage marker in patients with RA.

### Serum Gal-9 as a risk factor for high disease activity and functional limitations

**Figure 6:**
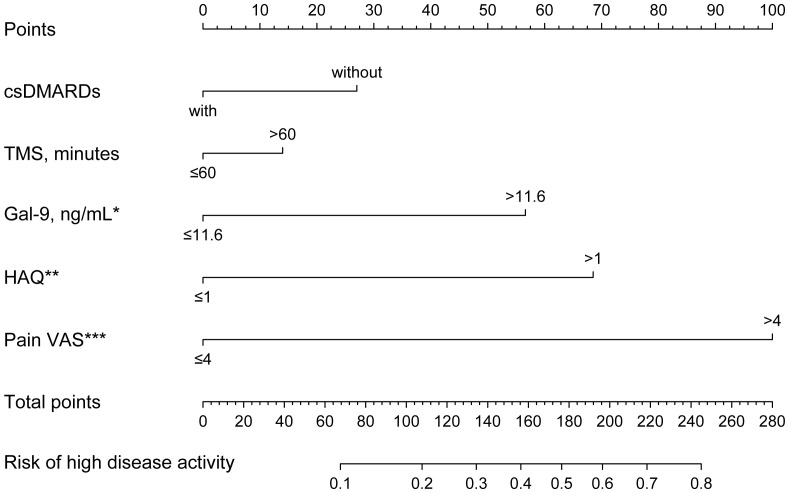
Nomogram to predict risk factors for high disease activity in patients with RA. Gal-9 >11.6 ng/mL, HAQ >1, and Pain VAS >4 are independent risk factors for high disease activity in RA. *P < 0.05, **P < 0.01, ***P < 0.001. Gal-9, galectin-9; HAQ, Stanford health assessment questionnaire disability index; Pain VAS, pain visual analogue scale; TMS, time of morning stiffness; bDMARDs, biological disease-modifying antirheumatic drugs. *P < 0.05, **P < 0.01, ***P < 0.001.

Serum Gal-9 concentrations in patients with RA with non-high disease activity were significantly lower than those with high disease activity (**Supplementary Figures S2A–D**). We used logistic regression analyses to explore whether serum Gal-9 levels could be a risk factor for high disease activity in patients with RA. Based on DAS28-CRP, there were 30.8% of patients with RA with high disease activity. Univariate logistic regression analysis showed that time of morning stiffness (TMS) >60mins, Pain VAS >4 (median Pain VAS), HAQ >1 and Gal-9 >11.6 ng/mL were positively associated with high disease activity in patients with RA, while treatment with csDMARDs was associated with a lower risk of high disease activity in patients with RA. Furthermore, the above five risk factors were applied to construct a nomogram to predict the independent risk of high disease activity in patients with RA. The result showed that Gal-9 >11.6 ng/mL (OR=3.138, 95% CI 1.150–8.567, p=0.026), HAQ >1 (OR=3.991, 95%CI 1.578–10.090, p=0.003) and Pain VAS >4 (OR=7.536, 95%CI 2.837–20.017, p <0.001) were independent risk factors for high disease activity in patients with RA (**Figure 6**; **Supplementary Table S1**).

**Figure 7:**
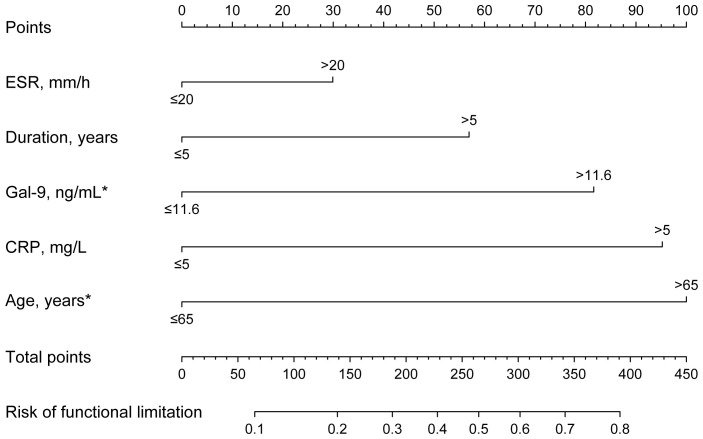
Nomogram to predict risk factors for functional limitations in RA. Functional limitation was defined as HAQ >1. The results show that Gal-9 >11.6 ng/mL and age >65 years are independent risk factors for functional limitations in RA. *P <0.05. Gal-9, galectin-9; CRP, C reactive protein; ESR, erythrocyte sedimentation rate.

Due to the significant associations between serum Gal-9 levels and functional limitations described above, logistic regression analyses were performed to evaluate the risk of functional limitations in patients with RA. Overall, 35.6% of patients with RA had functional limitations. Univariate logistic regression analyses showed that significantly higher odds of functional limitations (HAQ >1) were associated with age >65 years, disease duration >5 years, CRP >5 mg/L, ESR >20 mm/h, and Gal-9 >11.6 ng/mL. Notably, after adjusting for factors found to be significant in the univariate analyses, only age >65 years (OR=3.004, 95% CI 1.210–7.458, p=0.018) and Gal-9 >11.6 ng/mL (OR=2.455, 95%CI 1.017–5.926, p=0.046) were significantly correlated with an increased risk for functional limitations (**Figure 7**; **Supplementary Table S2**).

There were 85.6% of RA patients with advanced joint damage (stage II–IV). Univariate logistic regression analyses showed that age >65 years, disease duration >5 years, ESR >20 mm/h, CRP >5 mg/L and Gal-9 >11.6 ng/mL were positively associated with advanced joint damage. However, after adjusting for the above significant confounders, multivariate logistic regression analyses showed that Gal-9 >11.6 ng/mL was not an independent risk factor for advanced joint damage (**Supplementary Table S3**).

## Discussion

This study found that serum Gal-9 was significantly elevated in patients with RA compared with HCs. Patients with RA with higher levels of serum Gal-9 had worse disease characteristics, including higher core indicators of disease activity, more advanced functional limitations, and joint damage. A further multivariate regression analysis confirmed the association of Gal-9 and RA with high disease activity and functional limitations. These findings provide a comprehensive picture of the association of Gal-9 and the clinical index related to RA, especially with respect to functional limitations that have not been reported previously, and suggest that Gal-9 represents a potential marker in patients with RA.

Gal-9 is expressed in immune cells, endothelial cells, and synovial fibroblasts involved in the process of cell adhesion, differentiation, aggregation, and cell death (26). Gal-9 is also an apoptosis-inducing factor in malignancies and autoimmune diseases. In patients with multiple sclerosis, the Gal-9/Tim-3 interaction favors apoptosis of myelin basic protein-specific T lymphocytes, which correlates with reduced disease progression (27). Gal-9/Tim-3 pathway is also involved in inducing T cell apoptosis in a herpes simplex virus-induced Behcet’s disease mouse model (28). Previous studies reported that Gal-9 can regulate apoptosis of CD4^+^ T cells and synovial fibroblasts in patients with RA (8, 29, 30). Endogenous Gal-9 can protect against apoptosis and enhance viability of synovial fibroblast in patients with RA (29). Knockdown of Gal-9 alleviates the progression of arthritis in a collagen-induced arthritis (CIA) mouse model through the PI3K/AKT/mTOR pathway (31). In contrast, exogenous Gal-9 preferentially induces apoptosis and suppresses synovial fibroblast proliferation in patients with RA (30). Gal-9 has also been reported to decrease the levels of pro-inflammatory cytokines, including IL-17, IL-12, and IFN gamma in the joints of CIA mice (32). The role of Gal-9 in the pathogenesis of RA is still unclear and is possibly related to the different locations of Gal-9. In this study, patients with RA had higher serum Gal-9 levels compared to HCs, which is consistent with previously reported results (13, 33). Our results also showed that serum Gal-9 levels were elevated in patients with RA who were older, had longer disease duration, had double-positive ESR and CRP, and advanced joint damage. These results suggest that Gal-9 plays a pivotal role in the progression of RA.

Elevated Gal-9 levels have been observed in serum, synovial fluid, synovial tissue, and synovial fibroblasts of patients with RA (13, 14, 30). Previous studies have investigated the relationship between Gal-9 and RA disease activity, but results have been inconsistent. Wiersma et al. (12) reported that serum Gal-9 levels were elevated in patients with RA and positively correlated with disease activity, which is in line with the results of our study. Wang et al. (34) also reported that the plasma Gal-9 levels positively correlated with CRP, SDAI, and CDAI of patients with RA. In contrast, Ameen et al. (35) reported that serum Gal−9 levels were significantly and negatively correlated with DAS28-ESR. The discrepancy between these studies may be due to different inclusion criteria and even subgrouping methods of patients with RA. Morning stiffness is one of the classical manifestations of RA and is closely related to RA disease activity. We found that elevated Gal-9 levels were significantly related to prolonged morning stiffness. Furthermore, patients with RA having high disease activity also had higher serum Gal-9 concentrations compared to those with a low and moderate disease activity, especially defined by DAS28-CRP and CDAI scores. Furthermore, patients with Gal-9 levels ≤11.6 ng/mL had lower ESR, CRP, 28TJC, 28SJC, PtGA, PrGA, and Pain VAS, and serum Gal-9 levels were observed to be significantly correlated with these clinical indices. Importantly, multivariate regression analysis showed that a high level of Gal-9 is an independent risk factor for high disease activity in patients with RA. Altogether, these results suggest that Gal-9 is a potential marker of disease activity in patients with RA.

D2T RA usually represents higher disease activity and worse responses to treatment (36). Previous studies have reported that Gal-9 expression correlates with the therapeutic response in patients with RA (37, 38). In this study, patients with D2T RA had significantly higher serum Gal-9 levels than non-D2T patients with RA. The result coincided with that of Sun et al., who found that good RA responders had significantly lower Gal−9 expression in CD3^+^ and CD4^+^ T-cell subsets and lower plasma Gal-9 levels than those of poor responders (37). Furthermore, Gal-9 levels have also been found to be closely related to the rate of remission in patients with RA and are considered a factor predicting the time of the first remission. It found the first remission time for patients with Gal-9 of ≤4490 pg/mL (4 months, 95% CI 2.56–5.44 months) was shorter than that for patients with Gal-9 levels >4490 pg/mL (15 months, 95% CI 11.27–18.73 months) (log-rank, p=0.000) (38). In this study, we also found that serum Gal-9 levels were elevated in patients with RA requiring bDMARDs treatment, suggesting that Gal-9 is an indicator of refractory RA.

Tanikawa et al. reported that Gal-9 induced osteoblast differentiation through the CD44/Smad signaling pathway *in vitro* (39). They also found that Gal-9 interacted with lipid rafts and induced proliferation of human osteoblasts through phosphorylation of the c-Src/ERK signaling pathway (40). However, the results are inconsistent as the study by Moriyama et al. revealed the inhibitory effect of Gal-9 on osteoclastogenesis through Tim-3/Gal-9 system (41). Radiographic grading of hands is the most commonly used method for evaluating joint destruction (42). Few studies have described the relationship between Gal-9 levels and radiographic joint damage in RA. Matsumoto et al. reported serum Gal-9 levels were significantly higher in patients with RA with advanced joint damage (stage II–IV) compared to those without joint damage (stage I) (43). Fujita et al. found increasing serum levels of Gal-9 in patients with RA with progressive joint damage (stage II–IV) and with low titers of ACPA, but not with high titers of ACPA (13). In the present study, both the mTSS and the Steinbrocker radiographic stage were used to assess joint damage. The results revealed that patients with RA with radiographic joint damage (mTSS >0) or advanced joint damage (stage II–IV) had significantly higher serum Gal-9 levels compared to those without. Furthermore, patients with RA with Gal-9 >11.6 ng/mL had higher radiographic scores, including the mTSS, JSN, and JE subscores, and had a higher percentage of advanced joint damage. These results suggest that Gal-9 showed considerable value in identifying radiographic joint damage in RA.

The HAQ score reflects work capacity, household work performance, and the ability to live independently, and is a simple, effective, and accurate measure of physical function associated with activities of daily living (44). The HAQ score is associated with disease activity, joint destruction, quality of life-related to health, and psychosocial factors (45–49). Recent studies have shown that higher HAQ is an independent risk factor for D2T RA (50). These studies suggested the importance of HAQ in the assessment of RA in clinical practice. However, the association of Gal-9 with functional limitation of RA has never been reported. In this study, patients with RA with functional limitation had significantly higher serum Gal-9 levels than those without. In addition, we compared serum Gal-9 levels in different HAQ subgroups and found that most independent living deficits were associated with higher Gal-9 levels. In the Steinbrocker functional classification, we also found that the serum level of Gal-9 increased as the class of functional limitations increased. Furthermore, multivariate analysis in the present study showed that higher Gal-9 levels were significantly associated with functional limitations, suggesting that Gal-9 is a potential indicator of functional limitation in patients with RA.

There are several limitations to this study. First, this was a cross-sectional investigation and lacked evaluation of dynamic changes in Gal-9 levels during the progression of RA. Second, the number of patients from a single center was relatively small, and a larger longitude multicenter study is required to confirm our results and explore the pathogenesis of Gal-9 in RA.

In conclusion, Gal-9 may be considered a potential indicator for assessing and monitoring disease activity, functional limitations, and joint damage in RA. Further investigation is necessary on the pathological role of Gal-9 in the progression of RA.

## Data availability statement

The original contributions presented in the study are included in the article/**Supplementary Material**. Further inquiries can be directed to the corresponding authors.

## Ethics statement

The studies involving humans were approved by the Ethics Committee of the Shenzhen People’s Hospital. The studies were conducted in accordance with the local legislation and institutional requirements. The participants provided their written informed consent to participate in this study.

## Author contributions

JG: Data curation, Formal analysis, Methodology, Validation, Visualization, Writing – original draft, Writing – review & editing. XA: Data curation, Methodology, Validation, Visualization, Writing – original draft. BJ: Data curation, Formal analysis, Methodology, Writing – original draft. XZ: Data curation, Formal analysis, Methodology, Writing – original draft. LL: Data curation, Methodology, Writing – original draft. QH: Data curation, Methodology, Writing – original draft. JX: Data curation, Methodology, Writing – original draft. XH: Conceptualization, Funding acquisition, Supervision, Writing – review & editing. YC: Conceptualization, Data curation, Formal analysis, Funding acquisition, Investigation, Methodology, Project administration, Supervision, Writing – original draft, Writing – review & editing. DL: Conceptualization, Funding acquisition, Investigation, Project administration, Resources, Supervision, Validation, Writing – review & editing.

## Supplementary material

Supplementary Figure 1 Comparisons of RA disease activity indicators between Gal-9 subgroups. **(A)** ROC analysis showing the performance of Gal-9 in identifying RA patients with DAS28-CRP ≤ 5.1(non-high) from those with DAS28-CRP>5.1(High). **(B-H)** Comparisons of CRP, ESR, 28TJC, 28SJC, PtGA, PrGA, and Pain VAS between subgroups. Gal-9, galectin-9; CRP, C reactive protein; ESR, erythrocyte sedimentation rate; 28TJC, 28-joint tender joint count; 28SJC, 28-joint swollen joint count; PtGA, patient global assessment of disease activity; PrGA, provider global assessment of disease activity; Pain VAS, pain visual analogue scale.

Supplementary Figure 2 Elevated serum Gal-9 levels in RA patients with high disease activity. Comparison of serum Gal-9 levels between RA patients with non-high disease activity and individuals with high disease activity according to DAS28-CRP **(A)**, DAS28-ESR **(B)**, CDAI **(C)** and SDAI **(D)**. Gal-9, galectin-9; DAS28-CRP, disease activity score in 28 joints with four variables including CRP; DAS28-ESR, disease activity score in 28 joints with four variables including ESR; CDAI, clinical disease activity index; SDAI, disease activity was assessed with simplified disease activity index.
