# Supplementary material for: Galectin-9 as an indicator of functional limitations and radiographic joint damage in patients with rheumatoid arthritis
Source: Front Immunol. 2024 Jun 18;15:1419676. doi: 10.3389/fimmu.2024.1419676 (PMC11217821; doi:10.3389/fimmu.2024.1419676)
Supplement: Supplementary file 1 [file Table_1.docx]

Table S1 Univariate and multivariate logistic regression analyses for risk factors of high disease activity

| Variable |  | Univariate | | |  | Multivariate | | |
| --- | --- | --- | --- | --- | --- | --- | --- | --- |
|  |  | OR | 95% CI | P value |  | OR | 95% CI | P value |
| Age, years，>65 vs. ≤65 |  | 1.442 | 0.672-3.095 | 0.347 |  |  |  |  |
| Gender, female vs. male |  | 1.046 | 0.374-2.925 | 0.932 |  |  |  |  |
| Duration, years，>5 vs. ≤5 |  | 0.874 | 0.433-1.767 | 0.708 |  |  |  |  |
| Smoking, with vs. without |  | 0.730 | 0.188-2.835 | 0.650 |  |  |  |  |
| TMS, minutes, >60 vs. ≤60 |  | 2.867 | 1.253-6.558 | **0.013** |  | 1.326 | 0.435-4.048 | 0.620 |
| Pain VAS, >4 vs. ≤4 |  | 14.060 | 5.786-34.163 | **<0.001** |  | 7.536 | 2.837-20.017 | **<0.001** |
| HAQ, >1 vs. ≤1 |  | 8.435 | 3.816-18.647 | **<0.001** |  | 3.991 | 1.578-10.090 | **0.003** |
| Gal-9, ng/mL, >11.6 vs. ≤11.6 |  | 5.991 | 2.537-14.151 | **<0.001** |  | 3.138 | 1.150-8.567 | **0.026** |
| RF, positive vs. negative |  | 2.773 | 0.989-7.777 | 0.053 |  |  |  |  |
| ACPA, positive vs. negative |  | 1.611 | 0.690-3.762 | 0.270 |  |  |  |  |
| mTSS, >0 vs. =0 |  | 4.351 | 0.961-19.708 | 0.056 |  |  |  |  |
| bDMARDs, with vs. without |  | 2.141 | 0.726-6.320 | 0.168 |  |  |  |  |
| csDMARDs, with vs. without |  | 0.443 | 0.217-0.906 | **0.026** |  | 0.579 | 0.230-1.457 | 0.246 |

TMS: time of morning stiffness, Pain VAS: pain visual analogue scale, HAQ: Stanfordhealth assessment questionnaire disability index, Gal-9: galectin-9, RF: rheumatoid factor, ACPA: Anti-citrullinated protein antibody, mTSS score: Sharp/van der Heijde score, csDMARDs: conventional synthetic disease-modifying anti-rheumatic drugs, bDMARDs: biological disease-modifying anti-rheumatic drugs.
