# Supplementary material for: Galectin-9 as an indicator of functional limitations and radiographic joint damage in patients with rheumatoid arthritis
Source: Front Immunol. 2024 Jun 18;15:1419676. doi: 10.3389/fimmu.2024.1419676 (PMC11217821; doi:10.3389/fimmu.2024.1419676)
Supplement: Supplementary file 2 [file Table_2.docx]

Table S2 Univariate and multivariate logistic regression analyses for risk factors of functional limitations

| Variable |  | Univariate | | |  | Multivariate | | |
| --- | --- | --- | --- | --- | --- | --- | --- | --- |
|  |  | OR | 95% CI | P value |  | OR | 95% CI | P value |
| Age, years，>65 vs. ≤65 |  | 4.514 | 2.100-9.701 | **<0.001** |  | 3.004 | 1.210-7.458 | **0.018** |
| Gender, female vs. male |  | 1.032 | 0.384-2.772 | 0.951 |  |  |  |  |
| Duration, years，>5 vs. ≤5 |  | 3.189 | 1.544-6.586 | **0.002** |  | 1.871 | 0.824-4.249 | 0.135 |
| Smoking, with vs. without |  | 1.322 | 0.398-4.395 | 0.649 |  |  |  |  |
| TMS, minutes, >60 vs. ≤60 |  | 2.135 | 0.945-4.825 | 0.068 |  |  |  |  |
| ESR, mm/h, >20 vs. ≤20 |  | 3.406 | 1.217-9.533 | **0.020** |  | 1.390 | 0.385-5.018 | 0.615 |
| CRP, mg/L, >5 vs. ≤5 |  | 3.814 | 1.551-9.375 | **0.004** |  | 2.850 | 0.973-8.348 | 0.056 |
| Gal-9, ng/mL, >11.6 vs. ≤11.6 |  | 4.309 | 2.009-9.242 | **<0.001** |  | 2.455 | 1.017-5.926 | **0.046** |
| RF, positive vs. negative |  | 1.782 | 0.733-4.330 | 0.202 |  |  |  |  |
| ACPA, positive vs. negative |  | 0.931 | 0.432-2.007 | 0.854 |  |  |  |  |
| mTSS, >0 vs. =0 |  | 1.645 | 0.557-4.858 | 0.368 |  |  |  |  |
| bDMARDs, with vs. without |  | 1.232 | 0.413-3.677 | 0.709 |  |  |  |  |
| csDMARDs, with vs. without |  | 0.593 | 0.299-1.178 | 0.136 |  |  |  |  |

TMS: time of morning stiffness, ESR: erythrocyte sedimentation rate, CRP: C reactive protein, Gal-9: galectin-9, RF: rheumatoid factor, ACPA: Anti-citrullinated protein antibody, mTSS score: Sharp/van der Heijde score, csDMARDs: conventional synthetic disease-modifying anti-rheumatic drugs, bDMARDs: biological disease-modifying anti-rheumatic drugs.
