# Supplementary material for: Galectin-9 as an indicator of functional limitations and radiographic joint damage in patients with rheumatoid arthritis
Source: Front Immunol. 2024 Jun 18;15:1419676. doi: 10.3389/fimmu.2024.1419676 (PMC11217821; doi:10.3389/fimmu.2024.1419676)
Supplement: Supplementary file 3 [file Table_3.docx]

Table S3 Univariate and multivariate logistic regression analyses for risk factors of advanced joint damage.

| Variable |  | Univariate | | |  | Multivariate | | |
| --- | --- | --- | --- | --- | --- | --- | --- | --- |
|  |  | OR | 95% CI | P value |  | OR | 95% CI | P value |
| Age, years，>65 vs. ≤65 |  | 9.412 | 1.220-72.616 | **0.032** |  | 7.758 | 0.586-41.471 | 0.065 |
| Gender, female vs. male |  | 0.279 | 0.035-2.204 | 0.226 |  |  |  |  |
| Duration, years，>5 vs. ≤5 |  | 4.492 | 1.548-13.035 | **0.006** |  | 2.634 | 0.854-8.125 | 0.092 |
| Smoking, with vs. without |  | 1.930 | 0.236-15.783 | 0.540 |  |  |  |  |
| TMS, minutes, >60 vs. ≤60 |  | 2.742 | 0.602-12.494 | 0.192 |  |  |  |  |
| Pain VAS, >4 vs. ≤4 |  | 1.236 | 0.478-3.193 | 0.662 |  |  |  |  |
| HAQ, >1 vs. ≤1 |  | 1.928 | 0.663-5.607 | 0.228 |  |  |  |  |
| ESR, mm/h, >20 vs. ≤20 |  | 3.714 | 1.389-9.929 | **0.009** |  | 1.900 | 0.532-6.785 | 0.323 |
| CRP, mg/L, >5 vs. ≤5 |  | 4.222 | 1.622-10.992 | **0.003** |  | 2.787 | 0.868-8.944 | 0.085 |
| Gal-9, ng/mL, >11.6 vs. ≤11.6 |  | 2.902 | 1.095-7.693 | **0.032** |  | 1.226 | 0.394-3.811 | 0.725 |
| RF, positive vs. negative |  | 1.190 | 0.399-3.550 | 0.755 |  |  |  |  |
| ACPA, positive vs. negative |  | 0.429 | 0.119-1.546 | 0.196 |  |  |  |  |
| bDMARDs, with vs. without |  | 0.637 | 0.164-2.481 | 0.516 |  |  |  |  |
| csDMARDs, with vs. without |  | 0.509 | 0.185-1.398 | 0.190 |  |  |  |  |
