# Supplementary figures and images for: Galectin-9 as an indicator of functional limitations and radiographic joint damage in patients with rheumatoid arthritis
Source: Front Immunol. 2024 Jun 18;15:1419676. doi: 10.3389/fimmu.2024.1419676 (PMC11217821; doi:10.3389/fimmu.2024.1419676)

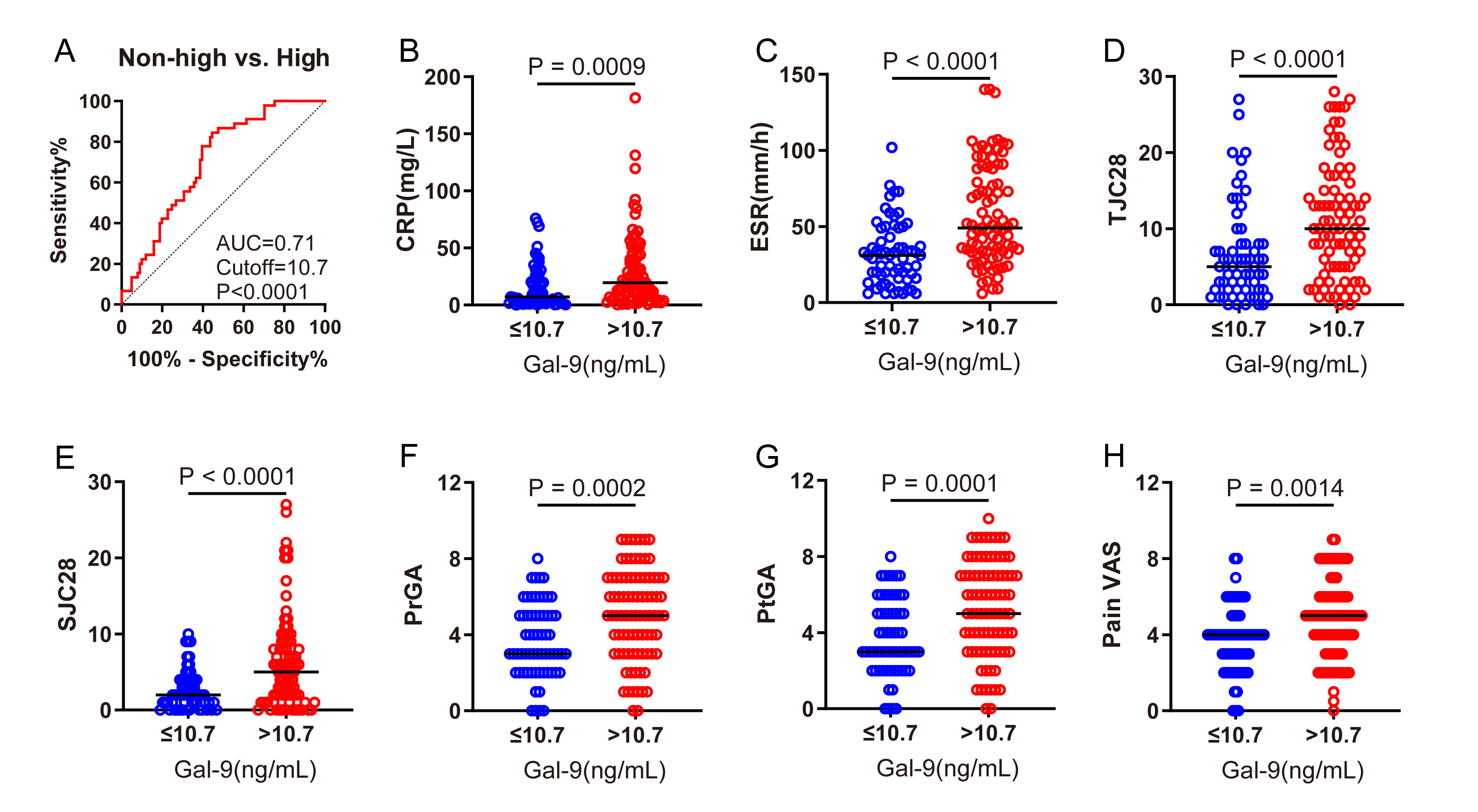

Supplement: Supplementary Figure 1 — Comparisons of RA disease activity indicators between Gal-9 subgroups. (A) ROC analysis showing the performance of Gal-9 in identifying RA patients with DAS28-CRP ≤ 5.1(non-high) from those with DAS28-CRP>5.1(High). (B-H) Comparisons of CRP, ESR, 28TJC, 28SJC, PtGA, PrGA, and Pain VAS between subgroups. Gal-9, galectin-9; CRP, C reactive protein; ESR, erythrocyte sedimentation rate; 28TJC, 28-joint tender joint count; 28SJC, 28-joint swollen joint count; PtGA, patient global assessment of disease activity; PrGA, provider global assessment of disease activity; Pain VAS, pain visual analogue scale. [file Image_1.tif]

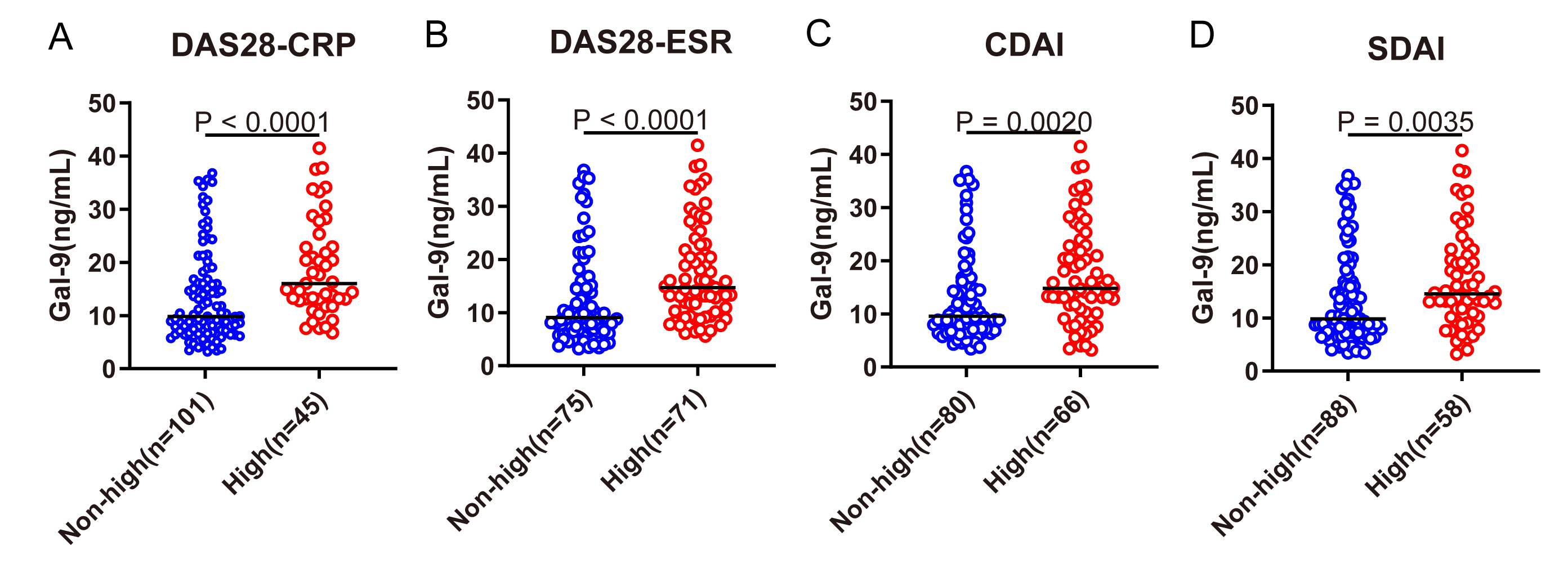

Supplement: Supplementary Figure 2 — Elevated serum Gal-9 levels in RA patients with high disease activity. Comparison of serum Gal-9 levels between RA patients with non-high disease activity and individuals with high disease activity according to DAS28-CRP (A), DAS28-ESR (B), CDAI (C) and SDAI (D). Gal-9, galectin-9; DAS28-CRP, disease activity score in 28 joints with four variables including CRP; DAS28-ESR, disease activity score in 28 joints with four variables including ESR; CDAI, clinical disease activity index; SDAI, disease activity was assessed with simplified disease activity index. [file Image_2.tif]
